# Outcomes of Common Major Surgical Procedures in Older Adults With and Without Dementia

**DOI:** 10.1001/jamanetworkopen.2020.10395

**Published:** 2020-07-14

**Authors:** Rebecca Masutani, Ajinkya Pawar, Hemin Lee, Joel S. Weissman, Dae Hyun Kim

**Affiliations:** 1 Department of Medicine, Beth Israel Deaconess Medical Center, Boston, Massachusetts; 2 Division of Pharmacoepidemiology, Department of Medicine, Brigham and Women’s Hospital, Boston, Massachusetts; 3 Center for Surgery and Public Health, Department of Surgery, Brigham and Women’s Hospital, Boston, Massachusetts; 4 Hinda and Arthur Marcus Institute for Aging Research, Hebrew Senior Life, Boston, Massachusetts

## Abstract

This cross-sectional study uses data from the Premier Healthcare Database to compare in-hospital mortality, home discharge, and hospital length of stay among older adults with and without dementia who have undergone major surgical procedures.

## Introduction

Dementia is associated with poor outcomes after surgical procedures.^1,2,3,4^ However, the types of major surgical procedures commonly performed in older adults with dementia and the surgical outcomes have not been characterized in a national sample. This cross-sectional study was conducted to identify commonly performed major surgical procedures in patients with dementia and compare in-hospital mortality, home discharge, and length of stay (LOS) among patients with and without dementia.

## Methods

We analyzed data from the Premier Healthcare Database, an all-payer, hospital-based, administrative and billing database that contains approximately 25% of inpatient admissions in the United States. We identified adults 65 years or older who underwent major surgical procedures, as defined by the Agency for Healthcare Research and Quality, between January 1, 2016, and March 31, 2018. Dementia was defined using the Chronic Conditions Data Warehouse algorithm.^5^ We estimated the odds ratios (ORs) and 95% CIs for in-hospital mortality and home discharge and the mean difference in LOS between patients with and without dementia using generalized estimating equations for logistic and linear regressions, respectively, to adjust for age, sex, race, admission source, Charlson Comorbidity Index, and procedure type and to account for clustering within hospitals. The outcome analysis was performed for the top 10 procedures combined and separately. Analyses were performed using SAS, version 9.4 (SAS Institute Inc). This study qualified for exemption from the Brigham and Women’s Hospital Institutional Review Board; the Premier Healthcare Database provided the researchers with a deidentified data set that did not have patient or hospital identifiers. The Partners Human Research Committee waived patient informed consent because this study was minimal risk, the waiver of informed consent did not increase the risk to human participants, and the research could not be practically conducted without a waiver of informed consent. This study followed the Strengthening the Reporting of Observational Studies in Epidemiology (STROBE) reporting guideline for cross-sectional studies.

## Results

The study population included 164 551 patients with dementia and 2 320 668 patients without dementia who underwent major surgical procedures at 804 hospitals. Patients with dementia were older (mean [SD] age, 81 [7] vs 74 [7] years), more likely to be women (57.4% vs 50.9%), more likely to be African American individuals (9.7% vs 7.0%), and more likely to have undergone an emergent operation (60.8% vs 25.8%) than those without dementia. Similar proportions of patients were treated at teaching hospitals (48.0% vs 47.3%) and urban hospitals (89.2% vs 89.8%). Of total surgical procedures, 6.4% were performed in patients with dementia (Table 1). The most commonly performed procedure was hip or femur repair (16.5%) in patients with dementia and knee arthroplasty (8.6%) in patients without dementia.

**Table 1.** Top 10 Most Commonly Performed Major Surgical Procedures in Patients With and Without Dementia^a^

Rank	Dementia (n = 203 121)	No. (%)	No dementia (n = 2 967 076)	No. (%)
1	Treatment for fracture or dislocation of hip and femur (CCS 146)	33 561 (16.5)	Knee arthroplasty (CCS 152)	254 152 (8.6)
2	Total or partial hip replacement (CCS 153)	20 094 (9.9)	Other surgical procedures on vessels other than head and neck (CCS 61)	184 355 (6.2)
3	Other surgical procedures on vessels other than head and neck (CCS 61)	13 813 (6.8)	Percutaneous transluminal coronary angioplasty with or without stent placement (CCS 45)	168 694 (5.7)
4	Other surgical therapeutic procedures on skin, subcutaneous tissue, fascia, and breast (CCS 175)	7957 (3.9)	Total or partial hip replacement (CCS 153)	165 350 (5.6)
5	Other therapeutic procedures (CCS 231)	7351 (3.6)	Other therapeutic procedures (CCS 231)	116 828 (3.9)
6	Percutaneous transluminal coronary angioplasty with or without stent placement (CCS 45)	7335 (3.6)	Other surgical therapeutic procedures on skin, subcutaneous tissue, fascia, and breast (CCS 175)	92 209 (3.1)
7	Amputation of lower extremity (CCS 157)	5515 (2.7)	Treatment for fracture or dislocation of hip and femur (CCS 146)	86 873 (2.9)
8	Gastrostomy: temporary or permanent (CCS 71)	4989 (2.5)	Spinal fusion (CCS 158)	81 778 (2.8)
9	Other surgical therapeutic procedures of urinary tract (CCS 112)	4491 (2.2)	Procedures on spleen (CCS 67)	81 285 (2.7)
10	Other fracture and dislocation procedures (CCS 148)	4051 (2.0)	Lens and cataract procedures (CCS 15)	66 445 (2.2)

Abbreviation: CCS, Clinical Classifications Software for *International Statistical Classification of Diseases, Tenth Revision*.

^a^ Data represent 203 121 procedures among 164 551 patients with dementia and 2 967 076 procedures among 2 320 668 patients without dementia from 804 hospitals. Some patients underwent more than 1 procedure.

After adjustment, patients with dementia experienced a higher incidence of in-hospital mortality (OR, 1.15; 95% CI, 1.10-1.20), a lower incidence of home discharge (OR, 0.30; 95% CI, 0.29-0.31), and increased LOS (mean difference, 2.35 days; 95% CI, 2.13-2.57 days) compared with patients without dementia undergoing the same procedures (Table 2). The highest odds of mortality were associated with genitourinary tract procedures (OR, 1.59; 95% CI, 1.31-1.91). The lowest odds of home discharge were associated with total or partial hip replacement (OR, 0.19; 95% CI, 0.18-0.21). Except for operations classified as other therapeutic procedures, the difference in LOS was the largest for breast and skin operations (mean difference, 2.59 days; 95% CI, 2.12-3.05 days).

**Table 2.** Outcomes of Top 10 Most Commonly Performed Major Surgical Procedures in Patients With Dementia Compared With Patients Without Dementia Undergoing the Same Procedures^a^

Major surgical procedure	OR (95% CI)		Length of stay, d, MD (95% CI)
	In-hospital mortality	Home discharge	
Treatment for fracture or dislocation of hip and femur (CCS 146)	1.16 (1.05-1.27)	0.66 (0.62-0.70)	0.24 (0.18-0.30)
Total or partial hip replacement (CCS 153)	1.41 (1.22-1.63)	0.19 (0.18-0.21)	1.21 (1.13-1.30)
Other surgical procedures on vessels other than head and neck (CCS 61)	1.01 (0.92-1.10)	0.36 (0.35-0.38)	2.49 (2.18-2.80)
Other surgical therapeutic procedures on skin, subcutaneous tissue, fascia, and breast (CCS 175)	1.37 (1.20-1.56)	0.32 (0.28-0.37)	2.59 (2.12-3.05)
Other therapeutic procedures (CCS 231)	1.15 (1.06-1.25)	0.32 (0.29-0.34)	3.81 (3.21-4.41)
Percutaneous transluminal coronary angioplasty with or without stent placement (CCS 45)	1.23 (1.13-1.35)	0.38 (0.36-0.40)	1.28 (1.13-1.44)
Amputation of lower extremity (CCS 157)	1.08 (0.90-1.29)	0.45 (0.42-0.49)	1.59 (1.23-1.94)
Gastrostomy: temporary or permanent (CCS 71)	0.83 (0.74-0.94)	0.61 (0.54-0.68)	0.83 (0.09-1.58)
Other surgical therapeutic procedures of urinary tract (CCS 112)	1.59 (1.31-1.91)	0.26 (0.24-0.28)	2.21 (1.92-2.51)
Other fracture and dislocation procedures (CCS 148)	1.46 (1.13-1.88)	0.41 (0.38-0.45)	1.28 (1.02-1.54)
Top 10 commonly performed procedures combined	1.15 (1.10-1.20)	0.30 (0.29-0.31)	2.35 (2.13-2.57)

Abbreviations: CCS, Clinical Classifications Software for *International Statistical Classification of Diseases, Tenth Revision*; MD, mean difference; OR, odds ratio.

^a^ The OR and MD were adjusted for age, sex, race, admission source, Charlson Comorbidity Index, and procedure type.

## Discussion

This study suggests that, in older patients with dementia, the risk of mortality, nonhome discharge, and prolonged hospitalization should be carefully weighed against the potential benefit of surgical procedures. Once the decision is made to proceed with surgical procedures, comanagement that includes both the geriatrics and the surgery departments can be beneficial.^6^ Our results can be useful to prioritize surgical procedures in which development of a comanagement program is necessary. Limitations of our study include reliance on diagnosis codes to identify dementia, which may capture only severe cases; lack of information on preadmission living situation, disease severity, and prognostic factors (eg, frailty) in administrative claims data; and unavailability of long-term outcomes beyond hospital discharge. It remains to be determined whether less severe dementia detected by a screening test is associated with poor postoperative outcomes. Nonetheless, our findings from a contemporary national sample provide useful information toward improving surgical care of older adults with dementia.
